# Mental, physical, and social well-being and quality of life in healthy young adult twin pairs discordant and concordant for body mass index

**DOI:** 10.1371/journal.pone.0294162

**Published:** 2023-12-06

**Authors:** Sakris K. E. Kupila, Bram J. Berntzen, Maheswary Muniandy, Aila J. Ahola, Jaakko Kaprio, Aila Rissanen, Kirsi H. Pietiläinen

**Affiliations:** 1 Obesity Research Unit, Research Program for Clinical and Molecular Metabolism, Faculty of Medicine, University of Helsinki, Helsinki, Finland; 2 Institute for Molecular Medicine Finland (FIMM), HiLIFE, University of Helsinki, Helsinki, Finland; 3 Folkhälsan Research Centre, Folkhälsan Institute of Genetics, Helsinki, Finland; 4 Abdominal Centre Nephrology, University of Helsinki and Helsinki University Central Hospital, Helsinki, Finland; 5 Obesity Centre, Endocrinology, Abdominal Centre, Helsinki University Hospital and University of Helsinki, Helsinki, Finland; Singapore General Hospital, SINGAPORE

## Abstract

**Objective:**

The relationship between obesity and mental health is complex and is moderated by the level of obesity, age, sex, and social and genetic factors. In the current study, we used a unique co-twin control design, with twin pairs discordant for body mass index (BMI), to control for shared genetic and environmental effects between obesity and several dimensions of mental health.

**Methods:**

We studied 74 monozygotic (MZ) twin pairs, of whom 36 were BMI-discordant (intra-pair difference in BMI ≥ 3 kg/m^2^), and 77 dizygotic (DZ) twin pairs (46 BMI-discordant). We assessed subjective health, especially mental health and mental well-being (depression, anxiety, self-esteem, health-related quality of life, life satisfaction, and social well-being) through questionnaires.

**Results:**

Heavier MZ co-twins from BMI-discordant pairs had poorer general health (58.8±3.0 vs. 72.4±3.8, *P* = 0.001, FDR = 0.017 on a scale from 0 to 100 where higher scores indicate more positive results), physical functioning (90.3±1.1 vs. 95.5±2.2, *P* = 0.024, FDR = 0.122), energy levels (55.6±3.4 vs. 66.6±3.3, *P* = 0.013, FDR = 0.109), and emotional well-being (65.9±3.2 vs. 75.4±2.9, *P* = 0.031, FDR = 0.122), as well as a tendency for depressive symptoms (8.4±1.3 vs. 5.6±0.9, *P* = 0.071, FDR = 0.166) compared to their leaner co-twins. Heavier DZ co-twins had poorer total physical well-being (91.6±1.9 vs. 95.6±1.0, *P* = 0.035, FDR = 0.356) and more depressive symptoms (4.3±0.9 vs. 2.4±0.5, *P* = 0.016, FDR = 0.345 on a scale from 0 to 63 where lower scores indicate fewer depressive symptoms) than their leaner co-twins. Association analyses, using all twin pairs, confirmed that higher BMI within pairs linked to general health, physical functioning and depressive symptoms. No association was found between BMI and anxiety, self-esteem, life satisfaction, or social well-being.

**Conclusions:**

In conclusion, this study underscores the notable association between elevated BMI and physical well-being and to a lesser extent between elevated BMI and depressive symptoms, while revealing no discernible connections with anxiety, self-esteem, life satisfaction, or social well-being.

## Introduction

Obesity is recognised as both a precipitant and a consequence of mental health problems [1–3]. On one hand, obesity is associated with stigma and prejudice, which may impair the mental health of persons with obesity [4, 5]. On the other hand, psychological problems may interfere with efforts towards weight control, resulting in the consumption of energy-dense foods and decreased physical activity and subsequently weight gain [6].

Mental health includes several different domains in our emotional, psychological, and social well-being. The most frequently studied domain in the field of obesity is depression. There is good evidence for prospective obesity-to-depression associations, while evidence for depression-to-obesity associations is less consistent [7]. However, not all studies have unequivocally found a positive relationship between obesity and future depression [8]. Fewer data are available on mood disorders other than depression, and most of these studies are cross-sectional. Some previous studies have found positive associations between anxiety and obesity [9–11], while others have not [12].

The life satisfaction, mental health -related quality of life, and self-esteem of adults with obesity compared to normal-weight adults remain less clear. Using the RAND 36-Item Health Survey, it has become evident that physical health -related quality of life is lower in individuals with obesity [13–15], but the findings on mental health -related quality of life are much less consistent [14–16]. As for self-esteem, its associations with Body Mass Index (BMI) in adults are unclear [17, 18].

Given that obesity and mental health are suggested to have shared genetic aetiology [3, 19–24], it would be beneficial to estimate how obesity is associated with psychological well-being without confounding genetic factors. Due to the significant heterogeneity of mental health measurements in previously published studies, it is difficult to obtain a global picture of how obesity and mental health are related. Additionally, because many studies include participants with severe obesity and significant life-impairing co-morbidities, less is known about the effects of less severe obesity in the absence of major comorbidities on mental health.

In this study, we take advantage of a rare set of well-characterised BMI-discordant and -concordant, healthy, young adult twin pairs to examine the associations between mental health and BMI. Monozygotic (MZ) twin pairs discordant for BMI are, despite their differing phenotype, completely matched for genetic variation, age, and sex. Additionally, same-sex dizygotic (DZ) twin pairs who are discordant for BMI share around 50% of their segregating genes but, similarly to the MZ twin pairs, have the same family environment, sex and age. This allows for research of BMI matching for genetic factors fully (in MZ pairs) or partially (in DZ pairs), as well as full adjustment for familial influences that confound studies comparing groups of unrelated leaner and heavier individuals. The aim of this study is to investigate the associations between an extensive profile of mental health factors (symptoms of depression and anxiety, self-esteem, health-related quality of life, life satisfaction, and social well-being) and BMI.

## Methods

### Participants

This cross-sectional study included 36 healthy MZ twin pairs discordant for BMI (within-pair difference ΔBMI ≥ 3 kg/m^2^; n = 28 men, n = 44 women, mean age 28.9±Standard Error (SE) 0.7 years) and 46 BMI-discordant DZ twin pairs (n = 50 men, n = 42 women, mean age 27.4±0.3 years). In addition, 38 MZ twin pairs concordant for BMI (ΔBMI < 3 kg/m^2^; n = 44 men, n = 32 women, mean age 29.4±0.6 years) and 31 BMI-concordant DZ twin pairs (n = 34 men, n = 28 women, mean age 28.3±0.4 years) were included as reference groups. All twin pairs were same-sex pairs. The BMI discordance cut-off point was determined in earlier studies [25, 26]. The pairs were identified from 2 population-based longitudinal studies of 10 complete Finnish birth cohorts, FinnTwin16 (years 1975–1979, n = 2578 pairs) and FinnTwin12 (years 1983–1987, n = 2839 pairs), with data retrieved between 2003 and 2013 [27]. All participants provided written informed consent. The Ethics Committee of the Helsinki University Central Hospital approved the study.

### Clinical assessments

Weight and height were measured to calculate BMI (weight in kilograms divided by height in meters squared (kg/m²)).

The twins were invited to an independent medical evaluation conducted by a physician. The evaluation included a medical history, physical examination, and laboratory assessments. This aimed to confirm the absence of concomitant diseases and disorders affecting weight or mood (i.e., anaemia, hypo- or hyperthyroidism) and regular medication (except contraceptives). Notably, twins with diagnosed psychiatric disorders, including eating disorders, were excluded.

### Mental health assessments

Depressive symptoms were assessed by the Beck Depression Inventory (BDI) [28]. A BDI score, with a range between 0 (no symptoms of depression) and 63 (severe symptoms of depression), was calculated for each participant using the principles described by Beck et al. [28]. The items on appetite and weight changes were amended to include both an increased appetite and increased weight instead of only decreased appetite and weight. Moreover, to separate intentional weight loss from weight loss as a symptom of depression, an additional item was added to the original 21 items of the BDI to assess any current attempt to lose weight (‘I try/don’t try to lose weight’). Accordingly, reported weight loss in a person with intentions to lose weight resulted in a score of 0, as opposed to an unintentional weight loss that was scored in the usual manner of the BDI [28]. The Cronbach’s alpha is 0.69.

Anxiety was assessed by the State–Trait Anxiety Inventory with separate indices for state and trait anxiety [29]. State anxiety refers to anxiety experienced when some threat is present, such as that induced by answering the inventory, whilst trait anxiety refers to general levels of feelings of anxiety in everyday life. Both a state and trait anxiety score, with a range between 20 (no anxiety) and 80 (high anxiety), were calculated for each participant as described by Julian et al. [29]. The Cronbach’s alpha is 0.89 for state and 0.94 for trait anxiety. Self-esteem was assessed by the Rosenberg Self-Esteem Scale with a summary scale from 0 to 30, where 30 is the best possible self-esteem, scored as in [30]. The Cronbach’s alpha is 0.95.

Health-related quality of life was assessed by the RAND 36-Item Health Survey 1.0 questionnaire and its eight internal health concepts (physical functioning, role limitations due to physical health problems, role limitations due to emotional problems, energy, emotional well-being, social functioning, pain, and general health) [31]. Items from the physical and mental concept areas were combined to create physical and mental summary scales. The summary scale for physical health-related quality of life included physical functioning, role limitations due to physical health problems, and pain, whereas the summary scale for mental health–related quality of life included emotional well-being and role limitations due to emotional problems. The rest of the components (energy, social functioning, and general health) covered both physical and mental dimensions and were thus not included in these summary scales. Each concept area and summary scale was scored as in [32]. The Cronbach’s alpha is 0.91.

Life satisfaction was assessed with Allardt’s four-item scale [33, 34] with items on overall feelings towards life (whether life is interesting, happy, and easy) and loneliness. These items were scored as described in [35] to form a scale from 4−20 (life satisfaction score 4−6 = satisfied, 7−11 = somewhat satisfied, 12−20 = dissatisfied). The Cronbach’s alpha is 0.77. Social well-being was assessed with another four items considering relationships with the co-twin, father, mother, and partner. These items were scored as described in [36] to form a satisfaction sum scale from 0 to 8 (ranging from 0 = not at all satisfied to 8 = fully satisfied). The Cronbach’s alpha is 0.83.

### Statistical analyses

Statistical analyses were performed with Stata 13.1 (Stata Corporation, College Station, TX, USA) and the R statistical computing environment (R Foundation for Statistical Computing) [37]. The number of twin pairs with answers from both twins to each scale in the questionnaire is shown in S1 Table. We applied non-parametric statistical tests due to the small sample and non-normally distributed variables in some of the measures. Comparisons between leaner and heavier co-twins (based on BMI) in both BMI-discordant and -concordant groups were performed using Wilcoxon signed-rank tests. We set the level of statistical significance to *P*<0.05 and provide exact p-values. Furthermore, because multiple testing increases the risk of type 1 errors, we calculated false discovery rate (FDR) adjusted p-values as described in [38].

We used the common language effect size, as described by McGraw in [39], to calculate the size of the within-pair differences. In this study, it indicates the probability that any given heavier co-twin of the particular pair in question scores higher on a mental health variable than the leaner co-twin. For example, in BMI-discordant MZ twin pairs, a common language effect size of 0.75 for depressive symptoms means a 75% chance that the heavier co-twin has greater depressive symptoms in any randomly chosen BMI-discordant MZ twin pair. Note that an effect size of 0.50 indicates that the leaner and heavier co-twins are equally likely to score higher on this particular mental health trait.

### Association analyses

Taking advantage of the twin design, we also examined the relationship between the heavier or leaner status of the co-twins with the outcome measures (only BMI-discordant pairs in both zygosities). We then investigated the relationship of the co-twins’ BMI difference as a continuous measure with the differences in outcome measures (all twin pairs in both zygosities). Both the above models utilized a linear mixed-model (R package *lmer* [40]) with clustering for family. Benjamini-Hochberg FDR corrected *P*<0.05 was considered significant.

Next, we tested to see whether the zygosity of the twin pairs influenced our results from the analysis outlined above. We used a linear mixed-model to test whether the within-twin relationship between BMI and the outcome measures differed between MZ and DZ twins. Benjamini-Hochberg FDR corrected *P*<0.05 was considered significant. In all association analyses, we used both the outcome measures as is and also scaled them to obtain z-values (standardized β) so that the results allowed us to directly compare the strength of relationships across all measures.

## Results

### Participant characteristics

The weight and height characteristics of the twins are summarised in Table 1. The heavier co-twins of the BMI-discordant MZ pairs weighed on average 17.9 kg (SE 1.3) more and had 5.9 kg/m^2^ (SE 0.4) higher BMI than their leaner twin-pair members. Similar intra-pair differences (Δ) in weight (22.6 kg, SE 1.8) and BMI (7.2 kg/m^2^, SE 0.5) were observed in the BMI-discordant DZ pairs.

**Table 1:** Basic characteristics of the leaner and heavier twin-pairs.

	MZ discordant (n = 72)		DZ discordant (n = 92)		MZ concordant (n = 76)		DZ concordant (n = 62)	
	Leaner	Heavier	Leaner	Heavier	Leaner	Heavier	Leaner	Heavier
Age (y)	28.9 (0.8)		27.4 (0.3)		29.4 (0.6)		28.3 (0.4)	
Height (cm)	172.0 (1.8)	172.4 (1.7)	173.3 (1.2)	174.8 (1.3)	173.0 (1.5)	173.2 (1.5)	173.4 (1.7)	171.8 (1.5)
Weight (kg)	76.2 (2.9)	94.1 (3.2)	65.0 (1.4)	87.6 (1.9)	73.4 (2.1)	77.2 (2.1)	71.0 (2.6)	74.6 (2.7)
BMI (kg/m^2^)	25.5 (0.8)	31.4 (0.9)	21.5 (0.4)	28.7 (0.6)	24.4 (0.5)	25.6 (0.5)	23.5 (0.6)	25.2 (0.7)

Data are presented as mean (standard error). MZ = monozygotic, DZ = dizygotic, BMI = body mass index, n = number of pairs.

The height differences between heavier and leaner twins were not significant in any type of pair, while weight and BMI differences were highly significant (all *P*<0.002).

### Depression, anxiety, and self-esteem

**Fig 1:**
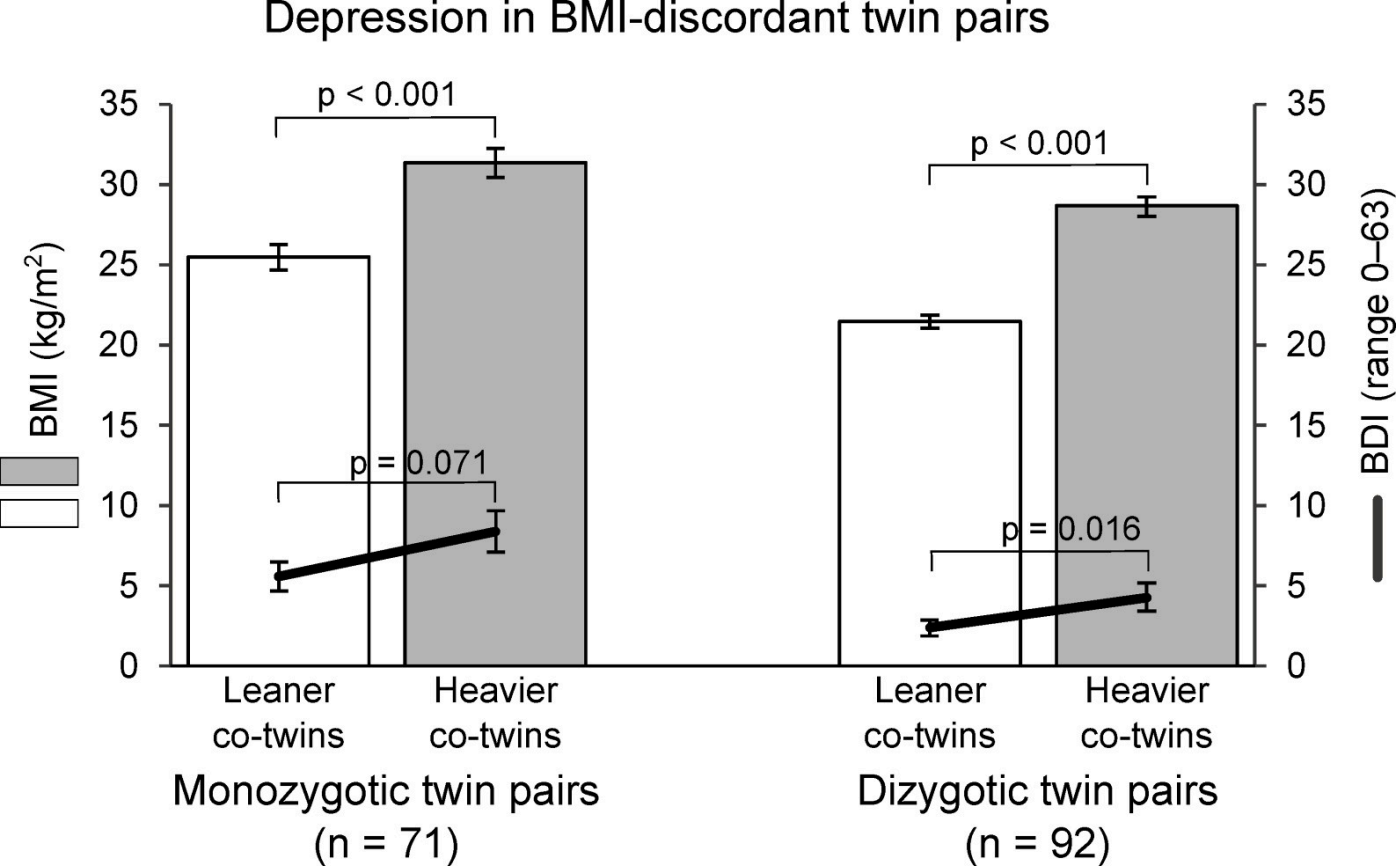
Mean Beck Depression Inventory (BDI) score in twin pairs discordant for Body Mass Index (BMI). The higher the BDI score, the more depressive symptoms.

We first assessed whether there were any differences in depressive symptoms (BDI) within the BMI-discordant twin pairs. We observed that the heavier co-twins of the BMI-discordant DZ twin pairs scored higher (4.3, SE 0.9) on the BDI questionnaire, indicating more depressive symptoms, than the leaner co-twins (2.4, SE 0.5; nominal *P* = 0.016, FDR = 0.345), and a similar tendency was observed within the BMI-discordant MZ twin pairs (*P* = 0.071, FDR = 0.166) (Fig 1 and Table 2). The common language effect size demonstrates that the probability of the heavier co-twins scoring higher on this questionnaire is 67% in MZ and 70% in DZ twin pairs. The BDI scores of the BMI-concordant co-twins were comparable (S2 Table).

**Table 2:** Mental well-being in leaner and heavier co-twins of monozygotic (MZ) and dizygotic (DZ) BMI-discordant pairs.

	MZ BMI-discordant (n = 72)					DZ BMI-discordant (n = 92)				
	Leaner	Heavier	*P*	FDR	%H>L	Leaner	Heavier	*P*	FDR	%H>L
**Beck Depression Inventory** ^**1**^										
Total score	5.6 (0.9)	8.4 (1.3)	0.071	0.166	67	2.4 (0.5)	4.3 (0.9)	0.016	0.345	70
**State-Trait Anxiety Inventory** ^**2**^										
State anxiety score	34.9 (1.4)	35.9 (1.6)	0.590	0.725	55	32.1 (1.0)	31.8 (1.0)	0.590	1.000	45
Trait anxiety score	34.9 (1.4)	37.0 (1.4)	0.130	0.234	64	33.4 (1.0)	33.3 (1.2)	0.960	1.000	50
**Rosenberg Self-Esteem Scale** ^**3**^										
Total score	23.1 (1.0)	22.0 (1.0)	0.590	0.725	45	25.1 (0.8)	24.7 (0.9)	0.900	1.000	51
**RAND 36-Item Health Survey 1.0** ^**4**^										
Physical functioning	95.5 (1.1)	90.3 (2.2)	0.024	0.122	27	99.0 (0.5)	96.0 (1.6)	0.130	0.835	38
Role limitations due to physical health	83.6 (5.6)	83.6 (6.0)	0.820	0.866	48	96.5 (2.0)	95.1 (2.4)	0.650	1.000	47
Role limitations due to emotional problems	78.2 (6.9)	75.9 (7.0)	0.900	0.866	49	96.3 (2.2)	90.7 (4.3)	0.440	1.000	45
Energy level	66.6 (3.3)	55.6 (3.4)	0.013	0.109	24	64.2 (2.7)	70.0 (3.0)	0.170	0.909	63
Emotional well-being	75.4 (2.9)	65.9 (3.2)	0.031	0.122	27	80.2 (2.0)	78.9 (2.3)	0.750	1.000	47
Social functioning	84.5 (4.0)	84.5 (3.8)	0.950	0.866	49	92.7 (2.0)	94.4 (2.7)	0.270	1.000	59
Pain	79.5 (3.8)	78.1 (4.1)	0.610	0.725	45	91.2 (1.5)	83.6 (3.4)	0.096	0.812	34
General health	72.4 (3.3)	58.8 (3.0)	0.001	0.017	17	78.9 (2.4)	75.3 (2.8)	0.260	1.000	39
Total physical well-being score	86.2 (2.8)	84.0 (3.4)	0.270	0.409	38	95.6 (1.0)	91.6 (1.9)	0.035	0.356	30
Total mental well-being score	76.8 (4.4)	70.9 (4.7)	0.210	0.356	37	88.7 (1.7)	84.7 (2.9)	0.390	1.000	42
**Life satisfaction** ^**5**^										
Life satisfaction score	8.2 (0.5)	9.3 (0.5)	0.059	0.165	67	7.6 (0.3)	7.2 (0.3)	0.300	1.000	40
**Relationship satisfaction** ^**6**^										
Relationship satisfaction score	3.9 (0.4)	3.7 (0.4)	0.290	0.409	40	3.7 (0.4)	3.9 (0.4)	0.920	1.000	49

Data are presented as mean (standard error). BMI = Body Mass Index. FDR = false discovery rate adjusted *P*-values. %H>L = probability on a scale of 0–100% that the heavier (H) co-twins score higher on a trait than the leaner (L) co-twins.

^1^ On a scale from 0 to 63, higher scores indicate more severe depressive symptoms.

^2^ On a scale from 20 to 80, higher scores indicate a higher degree of anxiety.

^3^ On a scale from 0 to 30, higher scores indicate higher self-esteem.

^4^ On a scale from 0 to 100, higher scores indicate higher quality of life.

^5^ On a scale from 4 to 20, higher scores indicate a higher level of dissatisfaction with life (score 4–6 = satisfied, 7–11 = intermediately satisfied, 12–20 = dissatisfied).

^6^ On a scale from 0 to 8, higher scores indicate a higher level of satisfaction with family relationships (mother, father, co-twin, partner; score 0 = from not at all to only somewhat satisfied, 8 = fully satisfied).

Leaner and heavier co-twins did not differ with respect to either anxiety or self-esteem in any of the twin groups.

### Quality of life, life satisfaction, and social well-being

**Fig 2:**
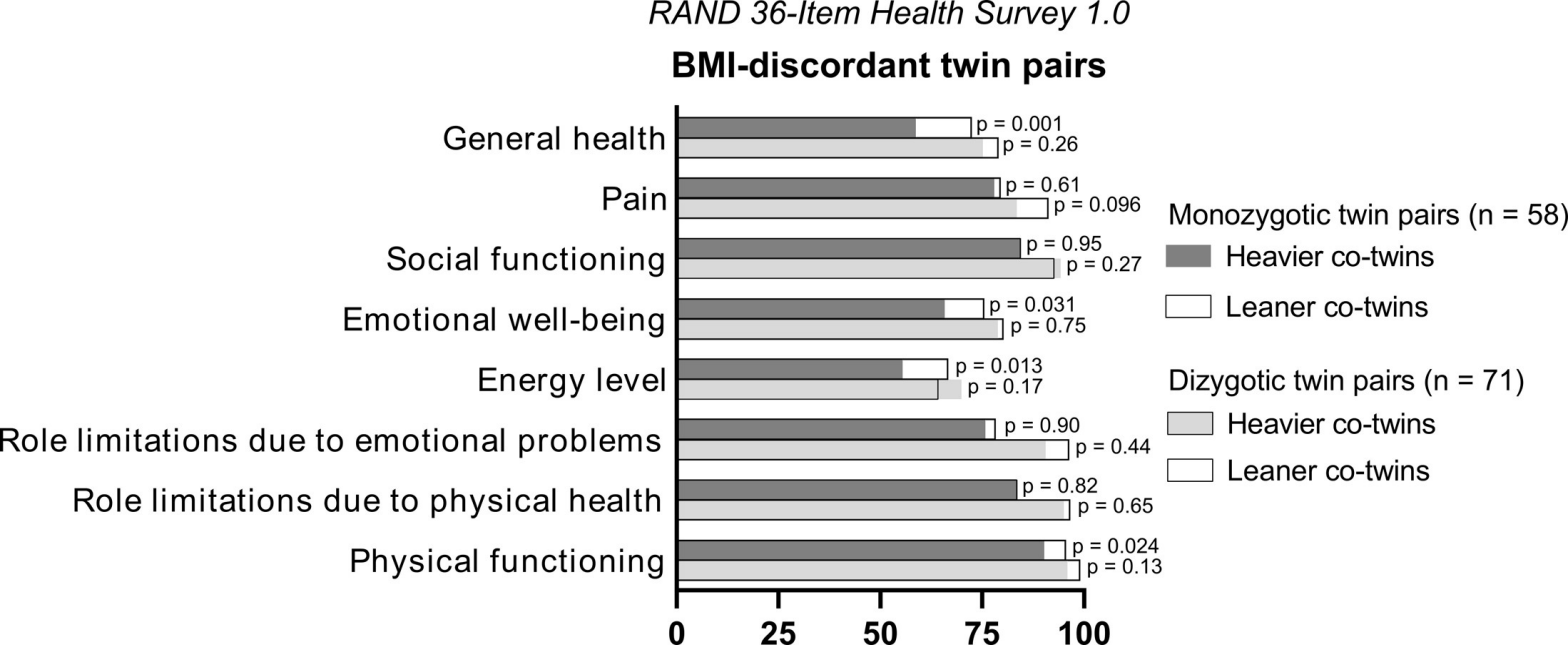
Dimensions of health-related quality of life (RAND 36-Item Health Survey 1.0) in twin pairs discordant for body mass index (BMI). Scores are presented as means. On a scale from 0 to 100, higher scores indicate higher quality of life.

When examining both physical and mental dimensions of health-related quality of life (RAND 36-Item Health Survey 1.0), we observed the following differences between the twin pairs: Heavier co-twins of BMI-discordant MZ twin pairs had worse physical functioning (*P* = 0.024, FDR = 0.122), felt less energetic (*P* = 0.013, FDR = 0.109), and reported lower emotional well-being (*P =* 0.031, FDR = 0.122) and general health (*P* = 0.001, FDR = 0.017) than their leaner co-twins (Fig 2). In BMI-discordant DZ (Table 2) and BMI-concordant MZ twin pairs (S2 Table), the overall physical well-being of the heavier group was worse than that of the corresponding leaner group. We observed no statistically significant differences within BMI-concordant DZ twin pairs. No groups had statistically significant co-twin differences in role limitations, social functioning, pain, or total mental well-being.

Lastly, we analysed life satisfaction and social well-being. Both BMI-discordant and -concordant MZ and DZ co-twins were comparably satisfied with their lives and family relationships (Table 2).

### Association analyses

We examined the relationship between the heavier or leaner status of the co-twins (BMI-discordant pairs, both zygosities) or within-pair difference in BMI as a continuous measure (all twin pairs) with the outcome measures (S3 Table). We found that the heavier co-twins had significantly lower scores for both physical function and general health. Using within-pair difference in BMI, rather than the heavy/lean status, we confirmed that physical function followed by general health had the strongest significant relationships with higher BMI within the co-twins. While both these measures were associated negatively with higher BMI, we also found a positive but weaker relationship between BDI score and higher BMI in the co-twins. Because co-twins are perfectly matched for age and sex, our results are free from these confounders. Additionally, we did not find any significant differences between MZ and DZ pairs when analysing the relationship of BMI to outcome measures.

## Discussion

In the current study, we analysed a wide range of psychological and quality of life questionnaire responses to obtain a global picture of how obesity is associated with mental well-being and mental health. We took advantage of a unique twin design, namely BMI-discordant MZ and DZ twin pairs, which allows for examining the relationships between excess body weight and mental health in leaner and heavier groups sharing the same (family) environment and matching either fully (MZ) or partially (DZ) for genetic factors. Twin pairs are also, by definition, matched for age, and in our study including only same-sex pairs are also matched for sex.

Our results show that heavier MZ co-twins from BMI-discordant pairs had poorer general health, physical functioning, energy levels, and emotional well-being, as well as a tendency for more depressive symptoms compared to their leaner co-twins. Association analyses, using all twin pairs, confirmed that higher BMI within pairs linked to general health, physical functioning, and depressive symptoms. However, we observed no significant associations between BMI and anxiety, self-esteem, life satisfaction, or social well-being.

We observed small differences in physical functioning between co-twins, consistently in the direction that might indicate a preliminary physical deterioration in an early stage of mild forms of overweight and obesity. This supports the findings of several previous studies which have reported that obesity is related to lower physical health–related quality of life [13–15].

Although the heavier co-twins of the BMI-discordant MZ twin pairs reported lower emotional well-being in the RAND 36-Item Health Survey 1.0, there were no statistically significant associations between BMI and the total mental well-being score. Similar results were found in studies by Ford et al. [13] with over 100,000 survey participants as well as Korhonen et al. [41] with almost 1200 healthy Finnish adults, where higher BMI was strongly associated with worse physical functioning but not with worse mental well-being.

The twin pairs had no or minimal symptoms of clinical depression. However, there was a trend towards higher BDI scores with higher BMI. This association between obesity and depression has been observed in several previous studies [12, 42, 43]. One possible explanation could be weight-related stigma. A person’s perceived self-value may decrease when internalizing negative attitudes and stereotypes related to higher body weight. Internalized weight stigma, and even just perceiving oneself as having overweight or obesity, has been shown to lead to both worse psychological well-being and lower self-esteem [44, 45], which in turn are risk factors for depression [46].

We observed no clinically meaningful differences between any of the co-twins in state, trait, or total anxiety measured by the State–Trait Anxiety Inventory. In an extensive survey of more than 62,000 participants by Scott et al. [10], anxiety was more prevalent in individuals with BMI > 35 kg/m^2^ (odds ratio 1.5). On the contrary, a later study of more than 120,000 participants by Bjørngaard et al. [43] suggested that general anxiety and obesity are not associated. Our study supports this latter finding.

We observed no association between BMI and self-esteem through the Rosenberg Self-Esteem Scale, similar to a study by Pearl et al. [18] on 240 individuals with obesity and binge eating disorder. On the contrary, a meta-analysis of 46 studies by Sikorski et al. [47] supports the general view that adults with obesity have lower self-esteem. As with all aspects of mental health, reduced self-esteem may also depend on the severity of obesity. In this study, the heavier twins in discordant DZ pairs had on average overweight (BMI 25−30) or, for MZ pairs, class I obesity (BMI 30−35). Additionally, the causal nature and direction of the association needs to be resolved.

All participants were comparably satisfied with their lives and family relationships, measured by Allardt’s four-item scale. Little is known about the associations between life satisfaction and obesity. Still, a cross-sectional study by Ul-Haq et al. [48] featuring over 16,000 participants suggests that men are more likely than women to report happiness when overweight or with slight obesity. Additionally, they found a significant negative correlation between obesity and happiness, which did not remain after the results were adjusted for self-reported health, suggesting that health rather than BMI mediated the association.

The current study has several strengths but also some limitations. Due to the extreme rarity of BMI-discordant MZ twin pairs, despite a comprehensive screening from two large nationwide twin populations, the number of twin pairs studied is quite low. This may especially lead to type 2 statistical errors (inability to find true positive associations) and a slightly increased risk for type 1 errors (false positives). Additionally, this study is cross-sectional and, therefore does not allow for definitive conclusions about causality. However, using BMI-discordant twin pairs enabled full control for sex, age, and family background between the leaner and heavier groups. We also matched the genotypic factors between the co-twins, fully in MZ and partially in DZ pairs. This design minimises the confounding of genetic factors and shared exposures and experiences with the observations. This is a major advantage for conclusions regarding such multifactorial traits as obesity and mental health.

In the future, prospective studies with larger cohorts and measured genotypes would better illuminate the associations between obesity and mental health factors. Given that acquired obesity is associated with poorer mental well-being, conducting mental health intervention studies targeting physical well-being would also be beneficial.

## Conclusions

Heavier MZ co-twins from BMI-discordant pairs had poorer general health, physical functioning, energy levels, and emotional well-being, as well as a tendency for more depressive symptoms compared to their leaner co-twins.

Association analyses, using all twin pairs, confirmed that higher BMI within pairs linked to general health, physical functioning, and depressive symptoms. No association was found between BMI and anxiety, self-esteem, life satisfaction, or social well-being.

## Supporting information

S1 Table Number of twin pairs with data available for each questionnaire. (PDF)

S2 Table Mental well-being in leaner and heavier co-twins of monozygotic (MZ) and dizygotic (DZ) BMI-concordant pairs. (PDF)

S3 Table Relationship of heavier-leaner status or BMI to outcome measures between co-twins. (PDF)
