# Supplementary material for: Mental, physical, and social well-being and quality of life in healthy young adult twin pairs discordant and concordant for body mass index
Source: PLoS One. 2023 Dec 6;18(12):e0294162. doi: 10.1371/journal.pone.0294162 (PMC10699637; doi:10.1371/journal.pone.0294162)
Supplement: S1 Table — (PDF) [file pone.0294162.s001.pdf]

S1 Table. Number of twin pairs with data available for each questionnaire.

	MZ BMI-discordant twin pairs (n = 72)	MZ BMI-concordant twin pairs (n = 76)	DZ BMI-discordant twin pairs (n = 92)	DZ BMI-concordant twin pairs (n = 62)
Beck Depression Inventory	71	75	92	61
State-Trait Anxiety Inventory	72	76	92	62
Rosenberg Self-Esteem Scale	71	72	91	61
RAND 36-Item Health Survey 1.0	58	55	71	59
Life satisfaction	58	57	72	62
Relationship satisfaction	72	76	92	62

MZ = monozygotic, DZ = dizygotic, BMI = Body Mass Index, n = number of pairs.
