# Supplementary material for: Mental, physical, and social well-being and quality of life in healthy young adult twin pairs discordant and concordant for body mass index
Source: PLoS One. 2023 Dec 6;18(12):e0294162. doi: 10.1371/journal.pone.0294162 (PMC10699637; doi:10.1371/journal.pone.0294162)
Supplement: S2 Table — (PDF) [file pone.0294162.s002.pdf]

S2 Table. Mental well-being in leaner and heavier co-twins of monozygotic (MZ) and dizygotic (DZ) BMI-concordant pairs.

	MZ BMI-concordant pairs (n = 76)				DZ BMI-concordant pairs (n = 62)			
	Leaner	Heavier	*p*	%H>L	Leaner	Heavier	*p*	%H>L
**Beck Depression Inventory[1]**								
Total score	4.0 (0.9)	3.9 (0.9)	0.73	47	3.8 (0.8)	3.6 (0.7)	0.91	51
**State-Trait Anxiety Inventory[2]**								
State anxiety score	32.3 (1.6)	33.1 (1.4)	0.48	57	32.8 (1.2)	31.5 (1.0)	0.73	46
Trait anxiety score	33.3 (1.8)	33.1 (1.7)	0.89	49	32.7 (1.3)	1.1 (1.1)	0.31	39
**Rosenberg Self-Esteem Scale[3]**								
Total score	24.7 (1.0)	25.3 (0.8)	0.51	56	24.5 (0.7)	25.2 (0.6)	0.35	60
**RAND 36-Item Health Survey 1.0[4]**								
Physical functioning	98.8 (0.5)	98.2 (0.6)	0.67	47	95.5 (2.6)	98.3 (0.6)	0.82	52
Role limitations due to physical health	94.6 (2.7)	83.9 (5.3)	0.070	34	87.5 (4.6)	96.4 (1.7)	0.11	64
Role limitations due to emotional Problems	91.7 (4.4)	90.5 (3.8)	0.46	44	85.6 (5.2)	93.3 (3.7)	0.20	61
Energy level	66.7 (2.9)	62.6 (4.3)	0.75	47	64.1 (3.3)	67.1 (2.6)	0.80	53
Emotional well-being	77.4 (2.7)	74.4 (3.2)	0.36	40	73.8 (3.5)	80.9 (2.1)	0.38	59
Social functioning	92.0 (3.0)	91.5 (2.7)	0.95	49	88.3 (3.6)	94.2 (2.1)	0.22	61
Pain	85.4 (3.9)	81.3 (3.5)	0.19	48	82.8 (4.6)	88.8 (2.3)	0.49	57
General health	74.6 (2.8)	77.0 (3.4)	0.39	59	80.3 (2.1)	80.3 (2.5)	0.98	50
Total physical well-being	92.9 (2.0)	87.8 (2.9)	0.039	28	88.4 (3.7)	94.7 (1.2)	0.097	68
Total mental well-being	84.4 (3.2)	82.2 (3.2)	0.31	39	79.7 (4.1)	87.1 (2.7)	0.38	59
**Life Satisfaction[5]**								
Life satisfaction score	7.3 (0.3)	8.2 (0.5)	0.20	64	8.1 (0.5)	7.2 (0.3)	0.18	36
**Relationship satisfaction[6]**								
Relationship satisfaction score	3.7 (0.5)	3.4 (0.5)	0.17	38	5.0 (0.3)	4.8 (0.4)	0.54	44

Data are presented as mean (standard error). %H>L = probability on a scale of 0 - 100% that the heavier co-twins score higher on a trait than the leaner co-twins.

[1] On a scale from 0 to 63, higher scores indicate more severe depressive symptoms.

[2] On a scale from 20 to 80, higher scores indicate higher degree of anxiety.

[3] On a scale from 0 to 30, higher scores indicate higher self-esteem.

[4] On a scale from 0 to 100, higher scores indicate higher quality of life.

[5] On a scale from 4 to 20, higher scores indicate a higher level of dissatisfaction with life (score 4–6 = satisfied, 7–11 = intermediately satisfied, 12–20 = dissatisfied).

[6] On a scale from 0 to 8, higher scores indicate a higher level of satisfaction with family relationships (mother, father, co-twin, partner; score 0 = from not at all to only somewhat satisfied, 8 = fully satisfied).
