# Supplementary material for: Mental, physical, and social well-being and quality of life in healthy young adult twin pairs discordant and concordant for body mass index
Source: PLoS One. 2023 Dec 6;18(12):e0294162. doi: 10.1371/journal.pone.0294162 (PMC10699637; doi:10.1371/journal.pone.0294162)
Supplement: S3 Table — (PDF) [file pone.0294162.s003.pdf]

S3 Table. Relationship of heavier-leaner status or BMI to outcome measures between co-twins.

	**Heavier-leaner status (36 BMI-discordant monozygotic twin pairs and 46 BMI-discordant dizygotic twin pairs)**					**BMI (74 monozygotic twin pairs and 77 dizygotic twin pairs, includes both BMI-discordant and BMI-concordant twins)**				
Measure	Twin pairs	Unstandardized ß-Values	Standardized ß-Values	P-value	FDR	Twin pairs	Unstandardized ß-Values	Standardized ß-Values	P-value	FDR
**Beck Depression**										
Total score	82	0.585	0.194	0.057	0.229	151	0.088	0.163	0.003	0.016
**State-Trait Anxiety**										
State anxiety score	81	0.264	0.034	0.803	0.894	149	0.081	0.050	0.386	0.441
Trait anxiety score	81	0.938	0.121	0.339	0.624	149	0.111	0.065	0.250	0.344
**Rosenberg Self-Esteem**										
Total score	81	-0.670	-0.115	0.351	0.624	147	-0.119	-0.107	0.062	0.199
**RAND 36-Item Health**										
Physical functioning	65	-4.000	-0.460	0.002	0.018	123	-0.529	-0.311	0.000	<0.001
Role limitations due to	65	-0.769	-0.033	0.838	0.894	123	-0.381	-0.083	0.203	0.325
Role limitations due to	65	-4.103	-0.136	0.439	0.702	123	-0.340	-0.061	0.338	0.416
Energy level	65	-1.641	-0.091	0.603	0.805	122	-0.115	-0.032	0.625	0.667
Emotional well-being	64	-4.883	-0.319	0.039	0.208	122	-0.348	-0.110	0.095	0.217
Social functioning	65	0.962	0.053	0.725	0.892	123	-0.076	-0.022	0.740	0.740
Pain	65	-4.808	-0.253	0.084	0.255	122	-0.460	-0.117	0.075	0.199
General health	65	-8.090	-0.455	0.001	0.017	123	-1.239	-0.364	0.000	<0.001
Total physical well-being	65	-3.192	-0.232	0.096	0.255	122	-0.454	-0.159	0.015	0.059
Total mental well-being	64	-4.650	-0.227	0.188	0.429	122	-0.348	-0.088	0.176	0.313
**Life satisfaction**										
Life satisfaction score	65	0.262	0.105	0.491	0.713	124	0.036	0.074	0.258	0.344
**Relationship**										
Relationship satisfaction	82	0.000	0.000	1.000	1.000	151	-0.037	-0.069	0.168	0.313

The table on the left shows how the heavier or leaner status in co-twins (only BMI-discordant twins) relate to the difference in outcome measure between the co-twins. Unstandardized ß-Values are regression coefficients and represent the change in the variable of interest when comparing heavier to leaner co-twins. A positive beta indicates an increase in the variable of interest when comparing heavier to leaner co-twins. Standardized ß-Values place all measures on the same scale (in z-score units) and

allow for comparison across the measures. Here, the heavier co-twins show lower physical functioning (4 unit [0.46 SD] decrease in the RAND 36-Item Health physical functioning scale) and general health (8 unit [0.46 SD] decrease in the general health scale) outcomes compared to their co-twins. The table on the right shows how 1 unit increase in BMI between co-twins (both BMI-concordant and BMI-discordant twins) relate to the difference in outcome measure between the co-twins. Unstandardized ß-Values are regression coefficients and represent the change in the variable of interest in relation to 1 unit increase in BMI. Here, 1 unit [1 SD] increase in BMI relates to lower physical functioning (0.53 unit [0.3 SD] decrease in the physical functioning scale) and general health (1.2 unit [0.36 SD] decrease in the general health scale) outcomes compared to their co-twins. Additionally, 1 unit [1 SD] increase in BMI relates to a higher score in the BDI questionnaire (0.09 unit [0.16 SD] compared to their co-twins). Benjamini-Hochberg FDR corrected $P<0.05$ is considered significant.
